# The expression profiles of immune genes in *Mus musculus* macrophages during *Staphylococcus aureus* infection

**DOI:** 10.1371/journal.pone.0190490

**Published:** 2018-01-05

**Authors:** Ziyan Wang, Fei Zhu

**Affiliations:** College of Animal Science and Technology, Zhejiang Agriculture and Forestry University, Hangzhou, China; University of Kansas Medical Center, UNITED STATES

## Abstract

*Staphylococcus aureus* is an important pathogen which is often the cause of major morbidity and mortality in both hospital and community settings. For this reason, we investigated the host cell early immune resoponse to *S*. *aureus* infection using genome-wide analysis. To do this, we infected *Mus musculus* RAW264.7 cells with *S*. *aureus* alone or in the presence of free peptidoglycan (PG), which appears in the *S*. *aureus* cell wall. Post infection, we performed a genome-wide analysis of RAW246.7 cells to identify significant changes in the gene expression profile. Further, we analyzed the infected RAW246.7 cells with transmission electron microscopy looking for the presence of bacterial cells inside the host cell. We also used flow cytometry to determine whether cells had induced apoptosis. The results showed that *S*. *aureus* induced apoptosis in the RAW246.7 cells but did not effectively clear away intracellular bacteria cells. However, *S*. *aureus* + PG treatment inhibited the apoptosis and activated the host cell inflammation response, possibly involving NF-κB and JAK-STAT pathways, as identified by genome-wide analysis, in RAW246.7 cells. Our study demonstrated for the first time that an independent application of free PG was capable of activating immune responses the host cells.

## Introduction

*Staphylococcus aureus* is an important nosocomial and community-acquired pathogen leading to human infections worldwide. Macrophages, part of the innate immune system, generally play a key role in host defense by recognizing, engulfing, and killing microorganisms [1]. However, *S*. *aureus* has developed an ability to escape the immune response of the host cells by inhibiting complement activation, blocking and destroying phagocytic cells and modifing host B cell and T cell responses [2]. A few studies have reported that *S*. *aureus* usually invade the host cells and persist intracellularly for varying periods of time in cell culture models [3–5]. Another study has reported that *S*. *aureus* can even survive in human epithelial cells for prolonged periods of time [6]. As a result, the survival of *S*. *aureus* within polymorphonuclear leukocytes might lead to prevention of macrophage efferocytosis and induce programmed necrosis [7]. Moreover, the intracellular persistence also provides *S*. *aureus* with an ideal strategy to escape professional phagocytes and promote recrudescent infection [4]. While interleukin-1 (IL-1) produced by phagocytes is an important cytokine orchestrating host defense against *S*. *aureus* [8], IL-1β is an important pro-inflammatory cytokine that activates monocytes, macrophages and neutrophils [9, 10]. Peptidoglycan (PG), a major component of the cell walls of gram-positive bacteria, can activate macrophages to phagocytose gram-positive bacteria through Toll-like receptor 2 (TLR2), which is recruited to phagosomes and discriminates between pathogens [11]. For phagocytosing *S*. *aureus*, PG must be particulated and internalized via phagocytosis [12]. However, this progress always be blocked by the components of *S*. *aureus*, so we used the free peptidoglycan to understand the early immune response against *S*. *aureus*. The peptidoglycan internalized via phagocytosis subsequently activates the NLRP3 inflammasome and IL-1β secretion [12]. We also found that nuclear factor kappa B (NF-κB) activation is required for phagocytosis of *staphylococcus aureus* by RAW264.7 cells [13]. As phagocytosis is a rapid and very important antimicrobial mechanism for host defense, it is vital to uncover the early interaction partners between pathogenic bacteria and phagocyte cells. This remains an active area of study as many of the details about the early interactions between *S*. *aureus* and macrophages are still not fully understood. To investigate the early immune response against *S*. *aureus* in macrophages, we applied free peptidoglycan (PG) in an effort to activate cytokine secretion in RAW 246.7 cells, a murine macrophage cell line, and performed a genome-wide analysis study. We compared host cell immune responses between *S*. *aureus* infection and PG treatment. We confirmed that *S*. *aureus* + PG treatment inhibited *S*. *aureus* mediated apoptosis and activated the host cell inflammatory response, involving the NF-κB and JAK-STAT pathways.

## Materials and methods

### Ethics statement

We did not use vertebrate animals, embryos or tissues in this study.

### Strain and cell culture

The *S*. *aureus* strain ATCC25923, widely used for medical assays, was obtained from Honghui Hospital (Hangzhou, China) and grown overnight in Columbia medium (Oxoid, the UK) with 2% NaCl at 37°C. All *Mus musculus* RAW264.7 cells (Cell Bank of Chinese Academy of Science, Shanghai, China) were cultured in RPMI 1640 media (Hyclone, Logan, USA) supplemented with 10% heat-inactivated fetal bovine serum (Gibco, USA), 10 mM HEPES (4-(2-hydroxyethyl)-1-piperazineethanesulfonic acid) (Invitrogen, USA), 0.11 mg/mL sodium pyruvate (Sigma, USA), 0.002 M L-glutamine (Sigma, USA) and pen/strep (1 mg/mL, 100 U/mL) (Invitrogen). To activate the phagocytic activity of RAW264.7 cells, PG (Sigma) was sonicated for 1 h and then added to each well at a concentration of 1 μg/mL, and then pre-incubated for 5 h before being used for the further investigation. RAW264.7 cells were infected with *S*. *aureus* alone or in combination with PG.

### Transmission electron microscopy

RAW264.7 cells were treated as described previously [14]. Sections of 100 nm thickness were cut in a Reichert Ultracut OMU3 microtome (Leica, Germany). This was followed by staining with uranyl acetate/70% methanol. Images were obtained using a Hitachi 7650 transmission electron microscope (Hitachi, Japan) operating at 70 kV.

### Microarray analysis

Total RNAs were extracted from either RAW264.7 cells (1×10^6^) as such (control), treated with *S*. *aureus* alone or treated with *S*. *aureus* in the presence of PG, using TRIzol reagent (Invitrogen) according to the manufacturer’s instructions. The total RNAs were then subjected to the Affymetrix Mouse Genome 430 2.0 array GeneChip (CapitalBio Corp., CA, USA) for gene expression analysis. The raw microarray data obtained were subsequently analyzed using Bio MAS (molecule annotation system) 3.0 software (Capital Bio Corp.). Differentially expressed genes were screened and clustered at a fold change ≥ 2 or ≤ 0.5 and p-value of ≤5%. Based on the DNA microarray data, the differently expressed genes were subsequently subjected to biological pathway analysis.

### Induction and measurement of apoptosis

To examine apoptosis, RAW264.7 cells were either incubated with *S*. *aureus* alone or *S*. *aureus* +PG for 12 h. Following these treatments, the cells were labeled with Alexa Fluor® 488 conjugated Annexin V and propidium iodide using an apoptosis kit (Invitrogen). Briefly, the cells were washed after harvesting and then suspended in a 900 μL cold Annexin-binding buffer containing 5 μL of Alexa Fluor® 488 conjugated Annexin V and 10 μL of propidium iodide solution (250 μg/ml). The cells were then incubated in this solution for 15 min at room temperature. Later the cells were analyzed by flow cytometry and fluorescent emissions were measured at 530 nm (FL-1) and >575 nm (FL-3) in triplicate.

### Quantitative real-time RT–PCR

Total RNAs were extracted from RAW264.7 cells (1×10^6^) with TRIzol reagent (Invitrogen) according to the manufacturer’s manual. Then the total RNAs were reverse transcribed to synthesize cDNAs using reverse transcriptase M-MLV (Takara, Japan). Quantitative real-time PCR was performed using gene-specific primers and TaqMan probes (S1 Table). Relative gene expression levels were calculated according to a method described previously [14], using *GAPDH* as an internal control. Real-time PCR amplification reactions were carried out in a final volume of 20 μL, which contained 10 μL Premix Ex Taq (TaKaRa), 1 μL diluted cDNA template, 7.2 μL dH_2_O, 0.3 μL of each TaqMan probe and 0.4 μL of each primer. PCR conditions were as follows, 95°C for 30 s, followed by 50 cycles of 95°C for 5 s and 60°C for 30 s.

### Statistical analysis

The data from three independent experiments were analyzed by one-way analysis of variance to calculate the mean and standard deviation of the triplicate assay. The significant differences between the treatments were determined using the Student’s t-test. Differences were considered to be significant at *P* < 0.05 and highly significant at *P* < 0.01.

## Results

### PG induced protection against *S*. *aureus*

**Fig 1:**
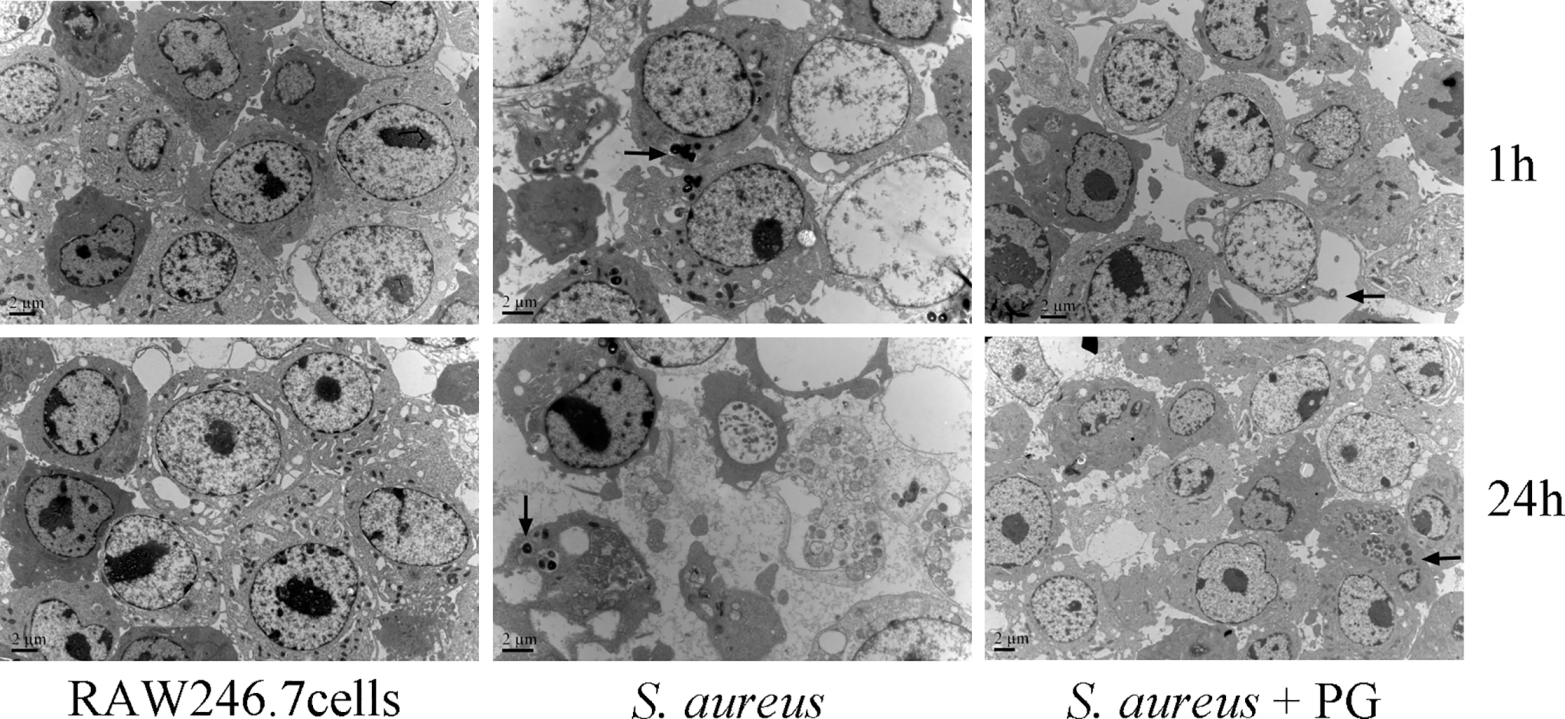
Transmission electron microscopy (TEM) analysis of RAW264.7 cells infected with *S*. *aureus*. The RAW264.7 cells were either not infected or infected with *S*. *aureus* alone or in combination with PG for two different time points. The upper panel shows the pictures by TEM after 1 h of *S*. *aureus* infection, while lower panel depicts the same after 24 h. Bar = 2 μm. Black arrow indicated the internalized *S*. *aureus*.

The protective effect of PG against *S*. *aureus* infection to the RAW264.7 cells was first analyzed by transmission electron microscopy (TEM). It was observed that the *S*. *aureus* treatment of RAW264.7 cells alone for 24 h resulted in their destruction and we observed large number of bacterial cells in the cytoplasm of RAW264.7 cells as indicated by the black arrow in Fig 1. Interestingly, 1 h incubation of RAW264.7 cells with *S*. *aureus* did not show any significant effect but we were still able to observe a few bacteria cells in their cytoplasm. In contrast, when *S*. *aureus*, bacterial cells were co-cultured with RAW264.7 cells, in the presence of PG, we observed a normal growth of RAW264.7 cells and there were only a few bacterial cells inside the cytoplasm even after an incubation period of 24 h. This data suggested that PG treatment induced an effective immune response against *S*. *aureus* infection in RAW264.7 cells.

### PG triggers immune response in RAW264.7 cells

**Fig 2:**
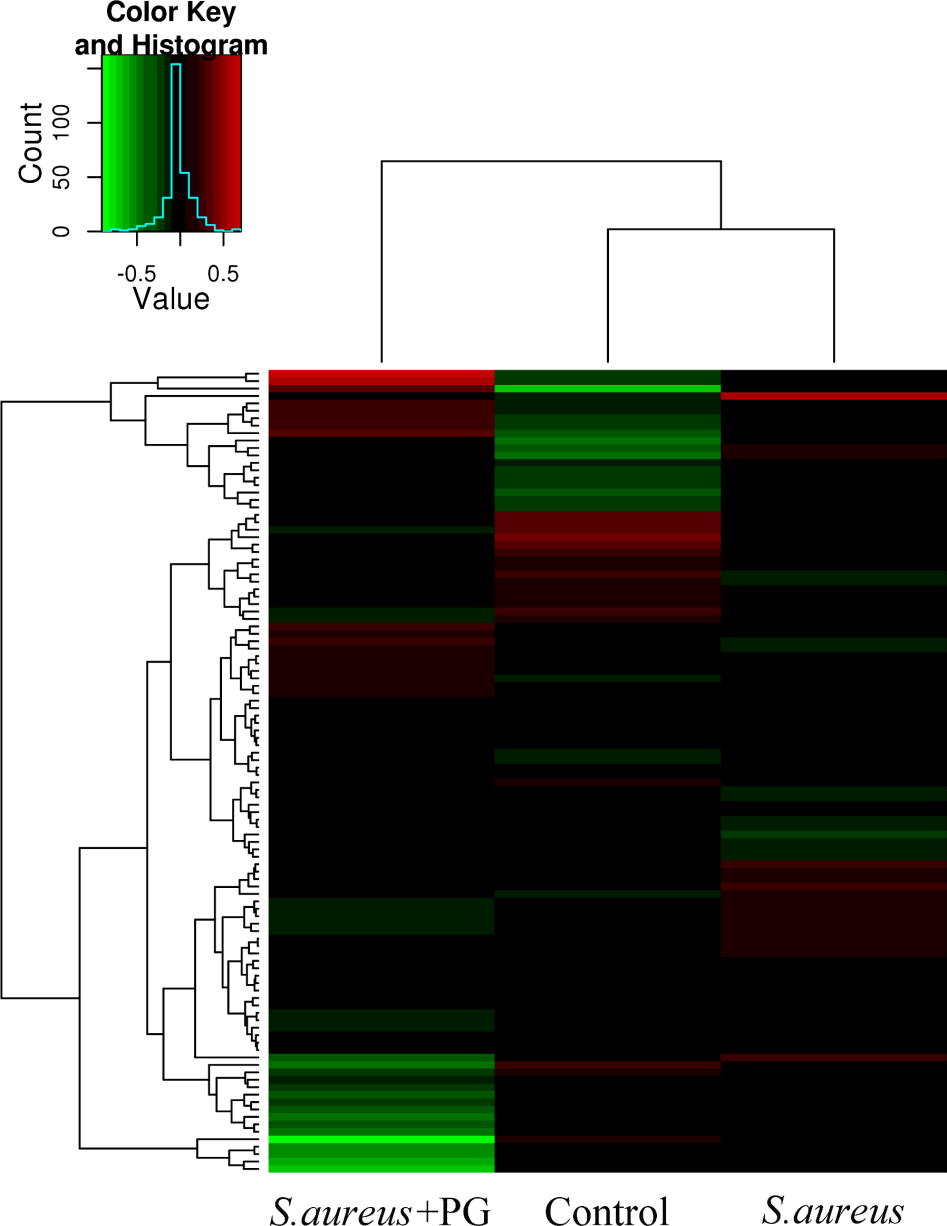
The gene expression profile microarray analysis. The gene expression profile analysis was performed using mouse DNA microarray chip using RNA isolated from RAW264.7 cells either treated with *S*. *aureus* or *S*. *aureus* + PG. Non-treated RAW264.7 cells were used as a control. Red signal indicates up-regulation of gene expression, while green signal depicts down-regulation.

Genome-wide analysis is a new approach that can be used to identify the host genes required for antibacterial immunity after specific treatment has been administered. In the current study, the genome-wide analysis of the *Mus* RAW264.7 cells in response to *S*. *aureus* infection was conducted using oligonucleotide microarray. The expression profiles of genes in the early response to *S*. *aureus* challenge showed that PG treatment altered the transcriptional response when compared with *S*. *aureus* alone and when compared with a no infection (control) as shown in Fig 2. Further detailed analysis revealed that *S*. *aureus* infection resulted in up-regulation of the expression levels of 6652 genes and down-regulation of 4257 genes (*P*<0.01), while *S*. *aureus* along with PG treatment significantly up-regulated 6588 genes and down-regulated 3796 genes (*P*<0.01). Following closer analysis of the data, it was observed that *S*. *aureus* and PG treatment together triggered many genes representing the host cell immune response and some other molecular pathways.

**Fig 3:**
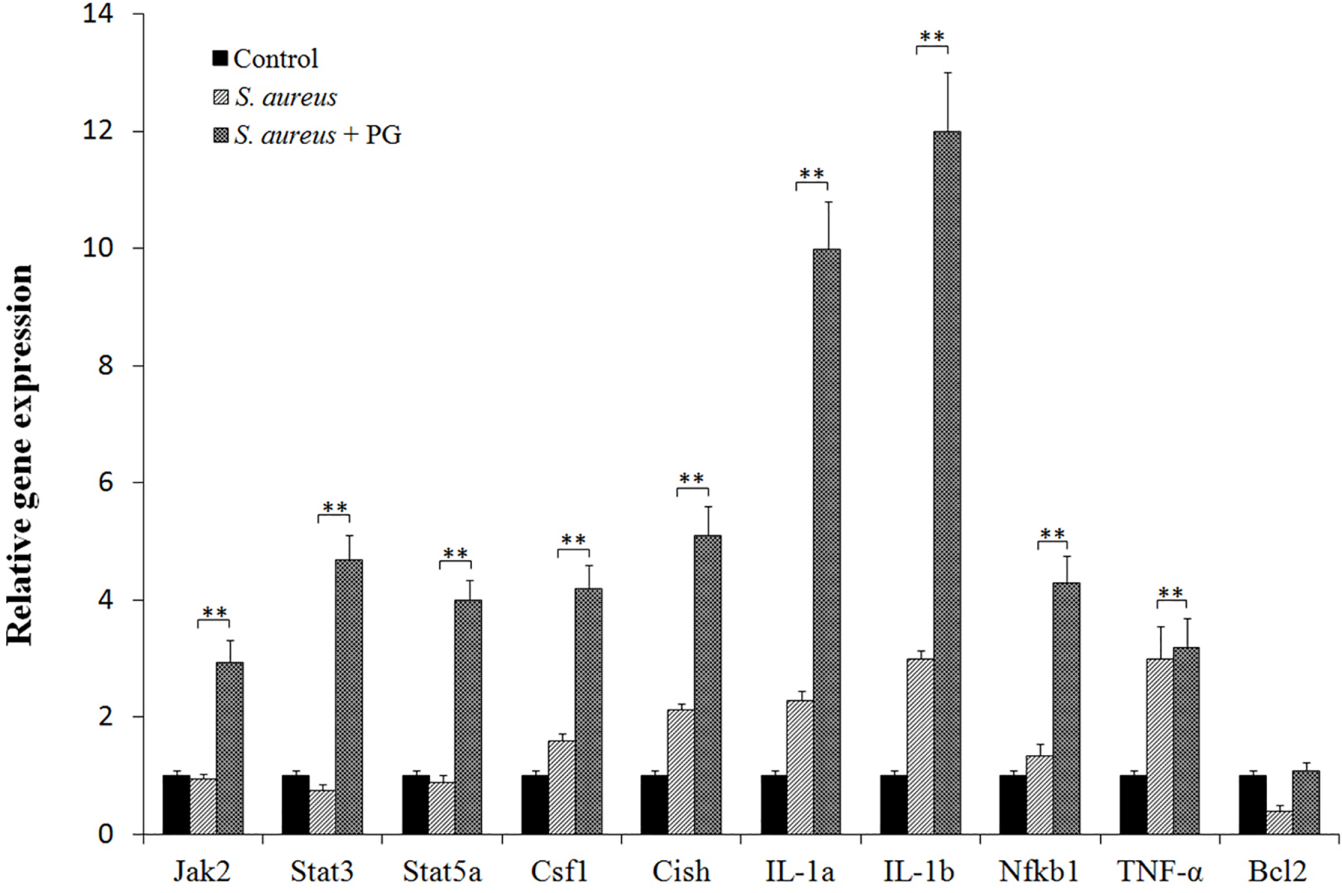
The expressions of important immune genes in RAW264.7 cells after the challenge. At 1 h after challenge with heat-inactivated *S*. *aureus* and heat-inactivated *S*. *aureus* + PG, the expression profiles of Jak2, Stat3, Stat5a, Csf1, Cish, IL-1a, IL-1b, Nfkb1, TNF-α and Bcl2 genes in RAW264.7 cells were characterized by quantitative real-time PCR. The *GAPDH* gene was used as a control. Statistically significant differences between treatments were indicated with asterisks (**, *P*<0.01).

Specifically, among the genes from the Toll pathway, the combination of *S*. *aureus* and PG pre-stimulation, resulted in the significant (*P*<0.05) upregulation of many genes like TLR1, TLR5, TLR9, Tollip, TIRAP and Pik3r5, as compared to *S*. *aureus* infection alone. In contrast, the expression levels of TLR3, TLR4 and Pik3r1 were significantly down-regulated (*P*<0.05) as shown in Table 1. Moreover, the expression levels of other important immune response related genes, including Il1a, Il1b, Bcl3, Il6, Il10, Il15, Nlrp3, Nfkb1, Nfkbiz, Nfkbie, Csf2, Csf3, Jak2, Stat3, Stat5a, Pik3r5, Cish and Socs3 were also significantly up-regulated in *S*. *aureus +PG* treatment at 1 h post incubation compared with *S*. *aureus* only (*P*<0.05, Table 2). Quantitative real-time RT–PCR analysis confirmed the same expression profile of these genes (Fig 3). Since, many of the up-regulated genes belong to the NF-κB and Jak-Stat pathways, it can be deduced that PG treatment might be playing an important role in activating these two pathways.

**Table 1:** Fold change in the expression profiles of the Toll pathway related genes induced by *S*. *aureus* and *S*. *aureus* +PG treatments.

Gene name	Description	Fold change (Mean ± SD)	
		*S*. *aureus*	*S*. *aureus* +PG
TLR1	toll-like receptor 1	0.70 ± 0.08	1.50 ± 0.21
TLR2	toll-like receptor 2	1.68 ± 0.35	1.71 ± 0.28
TLR3	toll-like receptor 3	0.79 ± 0.07	0.56 ± 0.07
TLR4	toll-like receptor 4	0.94 ± 0.12	0.49 ± 0.04
TLR5	toll-like receptor 5	0.47 ± 0.06	0.99 ± 0.15
TLR9	toll-like receptor 9	0.20 ± 0.03	1.72 ± 0.34
MyD88	myeloid differentiation primary response gene 88	1.20 ± 0.23	1.61 ± 0.34
CD14	CD14 antigen	1.52 ± 0.41	1.74 ± 0.39
Tollip	toll interacting protein	0.99 ± 0.18	1.93 ± 0.46
IRAK1	interleukin-1 receptor-associated kinase 1	0.95 ± 0.23	0.81 ± 0.21
IRAK4	interleukin-1 receptor-associated kinase 4	1.18 ± 0.25	0.94 ± 0.19
Traf6	TNF receptor-associated factor 6	1.76 ± 0.42	1.73 ± 0.57
TIRAP	toll-interleukin 1 receptor (TIR) domain-containing adaptor protein	1.09 ± 0.26	1.94 ± 0.63
Ticam1	toll-like receptor adaptor molecule 1	2.13 ± 0.71	2.50 ± 0.75
FADD	Fas (TNFRSF6)-associated via death domain	0.99 ± 0.16	1.01 ± 0.28
Rac1	RAS-related C3 botulinum substrate 1	1.05 ± 0.32	1.06 ± 0.22
Akt1	thymoma viral proto-oncogene 1	0.99 ± 0.16	0.92 ± 0.14
Pik3r1	phosphatidylinositol 3-kinase, regulatory subunit, polypeptide 1 (p85 alpha)	0.91 ± 0.23	0.46 ± 0.08
Pik3r5	phosphoinositide-3-kinase, regulatory subunit 5, p101	1.51 ± 0.57	3.13 ± 0.82

The numbers in the table refers to a fold change values compared to no treatment (control).

**Table 2:** Fold change in the expression profiles of some important immune function related genes induced by *S*. *aureus* and *S*. *aureus* + PG treatments.

Gene name	Description	Fold change (Mean ± SD)	
		*S*. *aureus*	*S*. *aureus* +PG
Il1a	Interleukin 1 alpha	9.21 ± 1.86	80.62 ± 6.65
Il1b	Interleukin 1 beta	32.27 ± 3.87	310.47 ± 18.62
Il6	Interleukin 6	8.46 ± 1.24	64.80 ± 4.49
Il10	Interleukin 10	3.01 ± 0.76	11.77 ± 2.13
Il15	Interleukin 15	0.79 ± 0.12	1.17 ± 0.26
Il18	Interleukin 18	1.11 ± 0.33	1.08 ± 0.27
Nlrp3	NLR family, pyrin domain containing 3	3.11 ± 0.85	4.77 ± 0.98
Nfkb1	nuclear factor of kappa light polypeptide gene enhancer in B-cells 1, p105	1.41 ± 0.43	3.88 ± 0.84
Nfkbiz	Nuclear factor of kappa light polypeptide gene enhancer in B-cells inhibitor, zeta	15.91 ± 1.96	25.59 ± 2.91
Nfkbie	Nuclear factor of kappa light polypeptide gene enhancer in B-cells inhibitor, epsilon	2.88 ± 0.78	3.83 ± 0.97
Csf1	colony stimulating factor 1	1.16 ± 0.22	1.20 ± 0.31
Csf2	colony stimulating factor 2	4.58 ± 0.93	104.69 ± 6.26
Csf3	colony stimulating factor 3	2.38 ± 0.65	59.76 ± 3.69
Jak1	Janus kinase 1	1.08 ± 0.27	1.38 ± 0.41
Jak2	Janus kinase 2	0.98 ± 0.21	2.86 ± 0.82
Stat3	signal transducer and activator of transcription 3	1.02 ± 0.26	2.71 ± 0.75
Stat5a	signal transducer and activator of transcription 5A	0.94 ± 0.23	2.17 ± 0.64
Pik3r5	phosphoinositide-3-kinase, regulatory subunit 5	1.51 ± 0.47	3.13 ± 0.89
Cish	cytokine inducible SH2-containing protein	2.38 ± 0.62	5.52 ± 0.91
Socs3	suppressor of cytokine signaling 3	2.59 ± 0.43	13.32 ± 2.51

The numbers in the table refers to a fold change values compared to no treatment (control).

Furthermore, we also looked at genes related to the apoptotic pathway and observed that *S*. *aureus* + PG treatment significantly up-regulated Bcl2, Bcl2l1, Bcl3, Casp4, TNF and Tnfrsf1b (*P*<0.05) expression, while significantly down-regulating the expression of Casp3, Casp7, Casp8, Casp9 and TRADD genes (*P*<0.05, Table 3).

**Table 3:** Fold change in the expression profiles of the apoptotic pathway related genes induced by *S*. *aureus* and *S*. *aureus* +PG treatments.

Gene name	Description	Fold change (Mean ± SD)	
		*S*. *aureus*	*S*. *aureus* +PG
Bcl2	B-cell leukemia/lymphoma 2	0.41 ± 0.05	0.99 ± 0.26
Bcl2l1	BCL2-like 1	0.69 ± 0.04	2.96 ± 0.68
Bcl3	B-cell leukemia/lymphoma 3	3.13 ± 0.89	6.13 ±1.37
Casp1	Caspase 1	0.97 ± 0.25	0.86 ± 0.22
Casp2	Caspase 2	0.63 ± 0.13	0.42 ± 0.04
Casp3	Caspase 3	1.88 ± 0.61	0.55 ± 0.06
Casp4	Caspase 4	1.47 ± 0.56	3.13 ± 0.75
Casp6	Caspase 6	0.88 ± 0.11	0.69 ± 0.08
Casp7	Caspase 7	0.86 ± 0.06	0.49 ± 0.05
Casp8	Caspase 8	1.12 ± 0.49	0.72 ± 0.12
Casp9	Caspase 9	0.97 ± 0.18	0.68 ± 0.09
TNF	tumor necrosis factor	9.05 ± 2.05	18.32 ± 2.62
Tnfrsf1b	tumor necrosis factor receptor superfamily, member 1b	1.27 ± 0.47	2.08 ± 0.78
TRADD	TNFRSF1A-associated via death domain	1.98 ± 0.55	0.77 ± 0.12

The numbers in the table refers to a fold change values compared to no treatment (control).

### PG antagonized *S*. *aureus*-induced apoptosis in RAW264.7 cells

**Fig 4:**
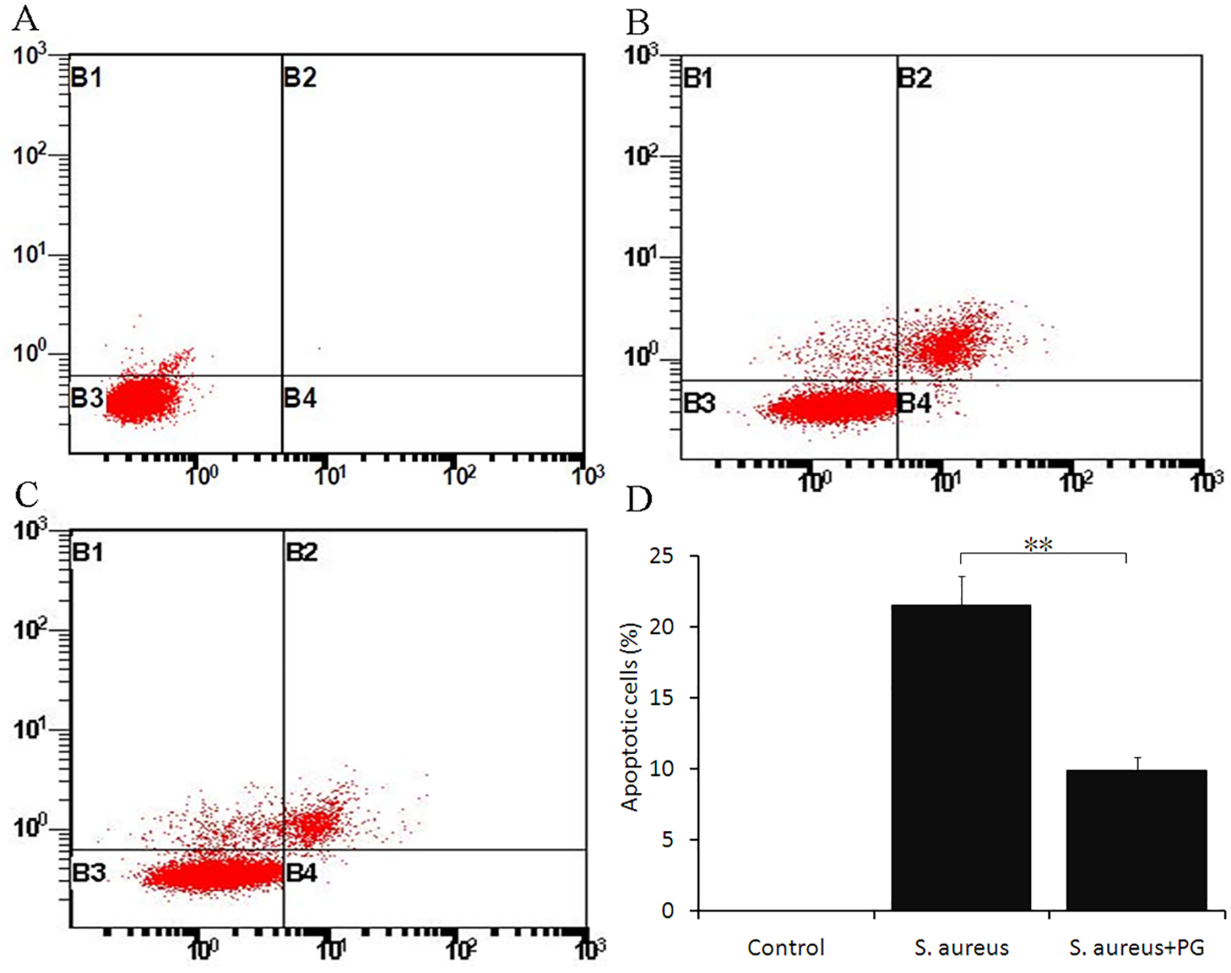
Flow cytometry based measurement of apoptosis in RAW264.7 cells, induced with *S*. *aureus* and *S*. *aureus* +PG treatment for 12 hours. Panel, A represents the apoptosis signal in the control cells. Panel, B represents the *S*. *aureus* induced apoptosis; and Panel, C depicts the *S*. *aureus* +PG induced apoptosis in RAW264.7 cells. Panel, D represents the mean percentage of apoptotic cells measured from three independent experiments. Double asterisks indicate a significant difference (*P*<0.01) between the samples.

In addition, we also analyzed the effect of PG on apoptosis induction by *S*. *aureus* through flow cytometric analysis. The data revealed that *S*. *aureus* induced apoptosis in RAW264.7 cells after 12 ho of treatment compared with the control cells. Interestingly, PG treatment resulted in the reversal of the *S*. *aureus* mediated apoptotic induction as shown in Fig 4A–4C. The percentage of apoptotic cells in *S*. *aureus* treatment were significantly higher than that in control cells (no infection and treatment), whereas *S*. *aureus* + PG treatment was able to significantly (*P*<0.01) inhibit the percentage of cells undergoing *S*. *aureus* induced apoptosis (Fig 4D). Moreover, the observation of significant (*P*<0.05) up regulation of the pro-apoptotic gene Casp3, and significant (*P*<0.05) down regulation of Bcl2, an anti-apoptotic gene, simultaneously in *S*. *aureus* treatment compared with *S*. *aureus* + PG as revealed by genome wide analysis, further suggested that *S*. *aureus* infection induced the apoptosis and the PG treatment, in turn antagonized this process.

## Discussion

The pathogenesis of *S*. *aureus* infection has continued to evolve and *S*. *aureus* has acquired the ability to anticipate and circumvent the host defense response [15]. It has been shown that *S*. *aureus* can escape the phagosomes of professional phagocytes and instead induce apoptosis and pyronecrosis in these cells [16]. The intracellular *S*. *aureus* also escaped the endosome and resulted in induction of apoptosis in epithelial cells [17]. In another study, it has been suggested that *S*. *aureus* suppressed the phosphorylation of nuclear factor-κB and thereby accelerated targeted cell death [18]. However, in this study, we have shown that PG stimulation promoted the expression of Nfkb1, Nfkbie, Nfkbiz, and other NF-κB pathway genes and that would eventually result in enhancement of *S*. *aureus* phagocytosis.

*S*. *aureus* biofilms have been shown to induce apoptosis in cells and tissues [19, 20]. Bcl-2, an integral membrane protein located mainly on the outer membrane of the mitochondria, is an important regulator of the programmed cell death pathways and can suppress apoptosis [21, 22]. Bcl3 can also promote cell proliferation and inhibit apoptosis [23, 24]. So, Bcl2, Bcl2l1 and Bcl3 are all anti-apoptotic proteins [25]. And TNF-alpha is able to activate the cell-death pathway [26, 27, 28]. In this context, our microarray expression analysis showed that PG stimulation up-regulated the expression of Bcl2, Bcl2l1, Bcl3, and TNF genes and this might explain the inhibition of *S*. *aureus*-induced apoptosis in the presence of PG which we observed.

PG treatment perhaps might have also provoked the inflammation response during the *S*. *aureus* challenge as inflammation related genes, IL-1α, IL-1β, Csf2, Csf3, Nfkb1 and TLR1, were all significantly up-regulated. The interleukin family has been closely linked to the innate immune response and the control of inflammation [29], and IL-1β is an important pro-inflammatory cytokine that has the potential to activate monocytes, macrophages and neutrophils [30]. IL-1β production by phagocytes is important for protection against the mucosal pathogen *S*. *aureus*. In order to activate its secretion, PG must be internalized via phagocytosis and subsequently activates the NLRP3 inflammasome and IL-1β secretion [12]. In our study, NLRP3 and IL-1β were also found to be very significantly up-regulated (*P*<0.01) by PG stimulation along with *S*. *aureus* infection compared with *S*. *aureus* infection alone. This indicated that the inflammation response was indeed activated by *S*. *aureus* + PG treatment, and it did play an important role in the immune response to *S*. *aureus* challenge.

In addition, *S*. *aureus*-induced corneal inflammation has been suggested to be mediated by TLR2 and myeloid differentiation factor 88 (MyD88) in resident epithelial cells and infiltrating neutrophils [31]. TLR2 mRNA expression in the liver, kidney, and lungs from postburn septic animals were rapidly up-regulated after *S*. *aureus* challenge [32]. Consistent with this observation, our results show upregulation of TLR2 by both *S*. *aureus* treatment alone or in combination with PG. However, *S*. *aureus* + PG treatment significantly upregulated MyD88 expression in comparison to *S*. *aureus* treatment alone. Also, *S*. *aureus* biofilms could circumvent TLR2 or TLR9-induced bacterial recognition pathways and significantly reduced IL-1β and TNF-α expression [33]. However, in the current study, it was observed that the presence of PG along with *S*. *aureus* resulted in upregulation of TLR1, TLR2, TLR9, MyD88, IL-1β and TNF-α expression, while *S*. *aureus* treatment alone could only up-regulate the expression of TLR2, IL-1β, and TNF-α at significantly lower levels. This difference in upregulation of certain genes in the presence of PG suggests that PG stimulation can effectively activate the inflammation and related immune response to *S*. *aureus* in Raw264.7 cells, while *S*. *aureus* alone circumvented TLR2-induced bacterial recognition pathways similar to *S*. *aureus* biofilms.

The Janus kinases (JAKs) play critical roles in several important intracellular signaling pathways, including the eponymous JAK/STAT pathway [34], central to the mediation of cytokine signaling [35, 36]. Almost 40 cytokine receptors signal through combinations of 4 JAK and 7 STAT family members, suggesting commonality across the JAK-STAT signaling system [37]. Similarly, the Janus family kinases (Jaks), Jak1, Jak2, and Jak3 have been shown to be involved not only in cell growth, survival, development, and differentiation of a variety of cells but are also critically important for immune cell function [38]. The JAK/STAT pathway played a critical role in the pro-inflammatory and apoptotic response in RAW264.7 cells [39]. Surprisingly, in our study we also observed that more than 18 key genes, including Jak2, Stat3, Stat5a, Pik3r5 and Cish, of JAK-STAT pathway were significantly up-regulated by *S*. *aureus* + PG infection, thus indicating that *S*. *aureus* + PG treatment might also activate JAK-STAT signaling.

Furthermore, PG from *S*. *aureus* has been shown to have an anti-apoptotic effect on keratinocytes and its effect seemed to be mediated by the production of the cellular inhibitor of apoptosis protein-2 [40]. This led to the escape of *S*. *aureus* from the endosomes/phagosomes and helped it proliferate within the cytoplasm, and eventually result in the death of host cells [41–44]. However, *S*. *aureus* may cover up the PG from it and prevent the immune response induced by PG. As PG plays an important role in *S*. *aureus* infection, we choose free PG extracted from *S*. *aureus* to activate immune response in RAW 246.7 cells before *S*. *aureus* infection. In this study, *S*. *aureus* was also able to escape from the endosomes/phagosomes and persist intracellularly in vacuoles for at least one day, ultimately resulting in Raw264.7 cell death. We show that added PG in advance can induce an effective immune response against *S*. *aureus* infection in RAW264.7 cells.

In summary, our results revealed that, although PG is a part of the cell wall structure of *S*. *aureus*, the independent application of free PG could exert different bioactivity. Free PG activated the complete immune response including inflammation to *S*. *aureus* infection and inhibited the apoptosis caused by *S*. *aureus*. Free PG may activate the JAK-STAT signaling pathway to regulate the inflammation and apoptotic response in the host cells. Based on previous studies, free PG can become a good strategy for clinical control of *S*. *aureus* infection. However, free PG treatment has its limitation for clinical therapy like the dosage and the drug-resistance strain. In addition, this is the first report suggesting the additional role of PG in *S*. *aureus* infection and this observation may have clinical importance.

## Supporting information

S1 Table Sequences of forward primer, reverse primer and TaqMan probe used for real-time quantitative RT-PCR. (DOC)

## Abbreviations

Bcl: B-cell lymphoma
Csf: colony stimulating factor
FBS: fetal bovine serum
GAPDH: glyceraldehyde-3-phosphate dehydrogenase
HEPES: (4-(2-hydroxyethyl)-1-piperazineethanesulfonic acid
Il: interleukin
Jak: Janus family kinases
MyD88: myeloid differentiation factor 88
Nfkb: nuclear transcription factor kappa-B
NLRP: NOD-like receptor protein
PG: peptidoglycan
STAT: Signal transducers and activators of transcription
TEM: transmission electron microscopy
TIRAP: Toll-interleukin 1 receptor adapter protein
TNF: tumor necrosis factor
TLR: Toll-like receptors
TRADD: Tumor necrosis factor receptor type 1-associated DEATH domain
