# Supplementary material for: The expression profiles of immune genes in Mus musculus macrophages during Staphylococcus aureus infection
Source: PLoS One. 2018 Jan 5;13(1):e0190490. doi: 10.1371/journal.pone.0190490 (PMC5755788; doi:10.1371/journal.pone.0190490)
Supplement: S1 Table — (DOC) [file pone.0190490.s001.doc]

**Table S1. Sequences of Forward Primer, Reverse Primer and TaqMan Probe Used for Real-time Q**uantitative RT-PCR

| **Gene** | **Forward (left) primer** | **Reverse (right) primer** | **TaqMan probe** |
| --- | --- | --- | --- |
| GAPDH | caatgtgtccgtcgtggatct | gtcctcagtgtagcccaagatg | cgtgccgcctggagaaacctgcc |
| Jak2 | cttccacatagacgagtcaacca | catcaagcagaggagcttcagc | atctgtaggttctgctgctgccact |
| Stat3 | ccgatgcctgtgggaagagtc | tgtcactacggcggctgttg | cctccagacggcagccacggca |
| Stat5a | gcagtcctggtgtgagaagc | tgagatgatgtccgtgatggtg | acctcagccagcatctcctccacg |
| Cish | ccctgcctatgtctaagcaagat | gccaccagacggttgatgac | cctagtgactcggtgctgcctatcc |
| Csf1 | cgctgcccttcttcgacatg | ccttcaggtgtccattcccaatc | cggctgctgctggtctgtctcctc |
| IL-1a | cgggaggagacgactctaaatatc | ggtcggtctcactacctgtg | tggcaactccttcagcaacacgggc |
| IL-1b | cctgggctgtcctgatgagag | tccacgggaaagacacaggtag | tcgcagcagcacatcaacaagagc |
| Nfkb1 | agtgcaaaggaaacgccagaag | gccagggcttccggtactc | tccgccaccgccactaccga |
| TNF-α | atgttgtagcaaaccctgaagct | attggccaggagggcatt | ctccaatggctgagccgacgtg^43^ |
| Bcl2 | gggctggggatgacttctctctcgc | ctgttgacgctctccacacac | tcgctaccgtcgtgacttcgcagag |

Nakata, M., Itou, T. ＆ Sakai, T. Quantitative analysis of inflammatory cytokines expression in peripheral blood mononuclear cells of the ferret (*Mustela putoriusfuro*) using real-time PCR. *Vet. Immunol. Immunopathol.* **130**, 88–91 (2009).
